# Supplementary material for: Who shares fake news on social media? Evidence from vaccines and infertility claims in sub-Saharan Africa
Source: PLoS One. 2024 Apr 9;19(4):e0301818. doi: 10.1371/journal.pone.0301818 (PMC11003631; doi:10.1371/journal.pone.0301818)
Supplement: S1 Table — This file provides the summary statistics of the study sample. (PDF) [file pone.0301818.s001.pdf]

Table S.1: Summary statistics

	Mean	SD	Min.	Max.	Obs.
Detection and sharing of misinformation					
Detection of fake news	0.55	0.50	0.00	1	5307
Intention to share	0.52	0.50	0.00	1	5307
Shared fake news	0.14	0.34	0.00	1	5307
Deliberate sharing intention	0.22	0.41	0.00	1	5307
Accidental sharing intention	0.30	0.46	0.00	1	5307
Deliberate sharing action	0.05	0.22	0.00	1	5307
Accidental sharing action	0.09	0.28	0.00	1	5307
Individual characteristics					
Age 18 - 29	0.62	0.49	0.00	1	5307
Age 30 - 39	0.27	0.44	0.00	1	5307
Age 40 - 49	0.08	0.27	0.00	1	5307
Age 50+	0.04	0.19	0.00	1	5307
Female	0.35	0.48	0.00	1	5307
Married	0.34	0.47	0.00	1	5307
No or primary education	0.02	0.14	0.00	1	5307
Secondary education	0.25	0.43	0.00	1	5307
Tertiary education	0.73	0.44	0.00	1	5307
(Self-)employed	0.54	0.50	0.00	1	5307
Rich	0.08	0.27	0.00	1	5307
Poor	0.17	0.38	0.00	1	5307
Cognitive skills	2.23	0.82	0.00	3	5307
Agreeableness	2.21	2.27	-6.00	6	5307
Openness	1.18	2.00	-6.00	6	5307
Risk taking	4.56	1.26	0.00	6	5307
Trust in institutions	2.10	0.59	0.00	3	5307
Social media: $<$ 1h last week	0.08	0.27	0.00	1	5307
Social media: 1-10h last week	0.50	0.50	0.00	1	5307
Social media: 11 - 20h last week	0.22	0.41	0.00	1	5307
Social media: $>$ 20h last week	0.20	0.40	0.00	1	5307
Vaccination	1.69	0.68	0.00	3	5307
Vaccine knowledge	1.02	0.62	0.00	2	5307
Vaccine hesitancy	-0.05	1.24	-1.84	4	5307

Note: The table reports the summary statistics of the main sample. Statistics included are the mean value, standard deviation, minimum and maximum value as well as the number of observations.
