# Supplementary material for: Who shares fake news on social media? Evidence from vaccines and infertility claims in sub-Saharan Africa
Source: PLoS One. 2024 Apr 9;19(4):e0301818. doi: 10.1371/journal.pone.0301818 (PMC11003631; doi:10.1371/journal.pone.0301818)
Supplement: S2 Table — This file provides the descriptive distribution of respondents per country. (PDF) [file pone.0301818.s002.pdf]

Table S.2: Observations by country

Country	Observations
Ghana	297
Kenya	3,170
Nigeria	639
South Africa	239
Tanzania	235
Uganda	699
Other	28
Total	5,307

Note: The table reports the number of observations by country.
