# Supplementary material for: Who shares fake news on social media? Evidence from vaccines and infertility claims in sub-Saharan Africa
Source: PLoS One. 2024 Apr 9;19(4):e0301818. doi: 10.1371/journal.pone.0301818 (PMC11003631; doi:10.1371/journal.pone.0301818)
Supplement: S3 Table — This file provides the regression results of individual characteristics associated with detecting misinformation. (PDF) [file pone.0301818.s003.pdf]

Table S.3: Detection of misinformation

	Detection of misinformation		Sharing intention
	(1)	(2)	(3)
	Binary measure	Continuous measure	
Age 30 - 39	0.040**	0.090**	0.024
	(0.017)	(0.037)	(0.017)
Age 40 - 49	0.052*	0.149**	0.056**
	(0.027)	(0.062)	(0.027)
Age 50+	0.058	0.185**	0.010
	(0.037)	(0.083)	(0.037)
Female	0.061***	0.087***	-0.129***
	(0.014)	(0.030)	(0.014)
Married	-0.031*	-0.062*	0.053***
	(0.016)	(0.035)	(0.016)
No or primary education	-0.044	-0.064	-0.009
	(0.047)	(0.109)	(0.048)
Secondary education	-0.059***	-0.112***	0.041***
	(0.016)	(0.035)	(0.016)
(Self-)employed	0.005	0.010	0.023
	(0.014)	(0.031)	(0.015)
Rich	-0.128***	-0.253***	0.059**
	(0.025)	(0.055)	(0.025)
Poor	-0.002	-0.021	0.013
	(0.018)	(0.039)	(0.018)
Cognitive skills	0.048***	0.104***	-0.056***
	(0.008)	(0.018)	(0.008)
Social media: $<$ 1h last week	-0.069***	-0.159***	0.016
	(0.025)	(0.054)	(0.025)
Social media: 11 - 20h last week	0.020	0.041	-0.001
	(0.017)	(0.037)	(0.017)
Social media: $>$ 20h last week	0.020	0.005	-0.025
	(0.018)	(0.039)	(0.018)
Agreeableness	0.002	0.002	-0.001
	(0.003)	(0.007)	(0.003)
Openness	0.003	0.009	-0.007**
	(0.003)	(0.008)	(0.003)
Risk taking	-0.014**	-0.015	0.017***
	(0.006)	(0.012)	(0.005)
Trust in institutions	-0.076***	-0.150***	0.063***
	(0.012)	(0.027)	(0.012)
Vaccination	-0.013	0.009	0.032***
	(0.010)	(0.022)	(0.010)
Vaccine knowledge	0.000	0.009	0.005
	(0.011)	(0.023)	(0.011)
Vaccine hesitancy	0.033***	0.014	-0.029***
	(0.006)	(0.013)	(0.006)
Observations	5,307	5,307	5,307
R^2	0.063	0.067	0.074

Note: The table reports OLS coefficient estimates and standard errors. Dependent variables are indicators that identify persons that correctly detected that misinformation was included in the article. In column 2 an alternative continuous measure is used for detection of misinformation that gives the extent to which a participants believes the report content is inaccurate on a 4-point Likert scale. Standard errors are robust. Regressions include vaccine-type, treatment assignment, and country fixed effects. ***,*,* denote significance at 1, 5 and 10%.
