# Supplementary material for: Who shares fake news on social media? Evidence from vaccines and infertility claims in sub-Saharan Africa
Source: PLoS One. 2024 Apr 9;19(4):e0301818. doi: 10.1371/journal.pone.0301818 (PMC11003631; doi:10.1371/journal.pone.0301818)
Supplement: S4 Table — This file provides the regression results of the main analysis using a probit instead of an OLS regression estimation. (PDF) [file pone.0301818.s004.pdf]

Table S.4: Alternative regression model: probit model

	Sharing action	Deliberate sharing	Accidental sharing
	(1)	(2)	(3)
Age 30 - 39	0.340***	0.308***	0.266***
	(0.057)	(0.076)	(0.065)
Age 40 - 49	0.471***	0.416***	0.375***
	(0.084)	(0.110)	(0.094)
Age 50+	0.529***	0.418***	0.451***
	(0.113)	(0.150)	(0.125)
Female	-0.208***	-0.049	-0.261***
	(0.049)	(0.064)	(0.057)
Married	-0.018	-0.125*	0.056
	(0.054)	(0.073)	(0.060)
No or primary education	-0.175	0.004	-0.289
	(0.166)	(0.199)	(0.204)
Secondary education	0.128**	0.091	0.116**
	(0.052)	(0.068)	(0.059)
(Self-)employed	0.178***	0.202***	0.117**
	(0.050)	(0.067)	(0.056)
Rich	0.113	-0.050	0.170*
	(0.081)	(0.118)	(0.089)
Poor	0.018	0.071	-0.028
	(0.061)	(0.081)	(0.071)
Cognitive skills	0.000	0.045	-0.031
	(0.028)	(0.037)	(0.032)
Social media: < 1h last week	-0.086	-0.188	-0.008
	(0.086)	(0.122)	(0.095)
Social media: 11 - 20h last week	-0.075	-0.063	-0.055
	(0.057)	(0.077)	(0.065)
Social media: > 20h last week	-0.084	-0.023	-0.104
	(0.060)	(0.078)	(0.069)
Agreeableness	0.002	-0.015	0.012
	(0.010)	(0.014)	(0.012)
Openness	-0.017	-0.029*	-0.005
	(0.011)	(0.015)	(0.013)
Risk taking	0.043**	0.063***	0.023
	(0.020)	(0.025)	(0.023)
Trust in institutions	0.097**	-0.052	0.177***
	(0.041)	(0.051)	(0.048)
Vaccination	0.063*	0.032	0.066*
	(0.034)	(0.045)	(0.038)
Vaccine knowledge	0.023	0.012	0.024
	(0.036)	(0.048)	(0.041)
Vaccine hesitancy	-0.032	0.015	-0.053**
	(0.020)	(0.025)	(0.023)
Observations	5,307	5,307	5,307
R^2	0.053	0.047	0.054

Note: The table reports coefficient estimates and standard errors of the probit regression of deliberate and accidental sharing actions on various individual characteristics as shown in the table. Regressions include vaccine-type, treatment assignment, and country fixed effects. Standard errors are robust. ***,*,* denote significance at 1, 5 and 10%.
