# Supplementary material for: Who shares fake news on social media? Evidence from vaccines and infertility claims in sub-Saharan Africa
Source: PLoS One. 2024 Apr 9;19(4):e0301818. doi: 10.1371/journal.pone.0301818 (PMC11003631; doi:10.1371/journal.pone.0301818)
Supplement: S5 Table — This file provides the regression results of the main analysis using alternative specifications of standard errors. (PDF) [file pone.0301818.s005.pdf]

Table S.5: Alternative regression model: clustered standard errors

	Sharing	Deliberate sharing	Accidental sharing
	(1)	(2)	(3)
Age 30 - 39	0.074***	0.032*	0.041*
	(0.007)	(0.009)	(0.011)
Age 40 - 49	0.111**	0.047**	0.064**
	(0.014)	(0.007)	(0.008)
Age 50+	0.123*	0.045	0.078
	(0.038)	(0.022)	(0.035)
Female	-0.042	-0.005	-0.037*
	(0.017)	(0.006)	(0.011)
Married	-0.003	-0.013	0.010
	(0.011)	(0.006)	(0.012)
No or primary education	-0.035	0.001	-0.036
	(0.038)	(0.020)	(0.021)
Secondary education	0.025	0.008	0.017
	(0.011)	(0.009)	(0.011)
(Self-)employed	0.036*	0.019*	0.017
	(0.011)	(0.005)	(0.008)
Rich	0.024	-0.004	0.028
	(0.018)	(0.008)	(0.016)
Poor	0.003	0.006	-0.003
	(0.006)	(0.004)	(0.008)
Cognitive skills	-0.000	0.004	-0.004
	(0.008)	(0.007)	(0.004)
Social media: $<$ 1h last week	-0.017	-0.016	-0.001
	(0.018)	(0.012)	(0.009)
Social media: 11 - 20h last week	-0.015	-0.007	-0.008
	(0.012)	(0.007)	(0.010)
Social media: $>$ 20h last week	-0.016	-0.001	-0.014
	(0.011)	(0.006)	(0.010)
Agreeableness	0.000	-0.002	0.002
	(0.003)	(0.001)	(0.003)
Openness	-0.004	-0.003	-0.001
	(0.003)	(0.001)	(0.003)
Risk taking	0.009	0.006	0.003
	(0.003)	(0.003)	(0.003)
Trust in institutions	0.020	-0.005	0.025**
	(0.008)	(0.007)	(0.004)
Vaccination	0.012	0.003	0.009
	(0.006)	(0.007)	(0.006)
Vaccine knowledge	0.004	0.001	0.003
	(0.008)	(0.003)	(0.008)
Vaccine hesitancy	-0.006	0.001	-0.007
	(0.003)	(0.002)	(0.004)
Observations	5,307	5,307	5,307
R^2	0.043	0.020	0.032

Note: The table reports coefficient estimates and standard errors from OLS regressions. Regressions include fixed effects for vaccine-type, treatment assignment, and country. Standard errors are clustered at the country-vaccine-type level. ***,*,* denote significance at 1, 5 and 10%.
