# Supplementary material for: Who shares fake news on social media? Evidence from vaccines and infertility claims in sub-Saharan Africa
Source: PLoS One. 2024 Apr 9;19(4):e0301818. doi: 10.1371/journal.pone.0301818 (PMC11003631; doi:10.1371/journal.pone.0301818)
Supplement: S6 Table — This file provides the regression results of the main analysis with a sub-sample that reports very good or good English language skills. (PDF) [file pone.0301818.s006.pdf]

Table S.6: Robustness check: Sub-sample with good English language skills

	Sharing	Deliberate sharing	Accidental sharing
	(1)	(2)	(3)
Age 30 - 39	0.072***	0.032***	0.040***
	(0.013)	(0.009)	(0.011)
Age 40 - 49	0.109***	0.049***	0.060***
	(0.022)	(0.015)	(0.018)
Age 50+	0.125***	0.046**	0.080***
	(0.031)	(0.020)	(0.026)
Female	-0.039***	-0.005	-0.033***
	(0.010)	(0.006)	(0.008)
Married	-0.003	-0.014*	0.011
	(0.012)	(0.008)	(0.010)
No or primary education	-0.046	-0.003	-0.042*
	(0.032)	(0.023)	(0.024)
Secondary education	0.023**	0.008	0.015
	(0.011)	(0.007)	(0.009)
(Self-)employed	0.036***	0.020***	0.016*
	(0.010)	(0.007)	(0.008)
Rich	0.020	-0.002	0.022
	(0.019)	(0.011)	(0.016)
Poor	0.006	0.007	-0.001
	(0.013)	(0.009)	(0.010)
Cognitive skills	0.001	0.004	-0.004
	(0.006)	(0.004)	(0.005)
Social media: $<$ 1h last week	-0.017	-0.016	-0.001
	(0.018)	(0.011)	(0.015)
Social media: 10 - 20h last week	-0.016	-0.007	-0.009
	(0.012)	(0.008)	(0.010)
Social media: $>$ 20h last week	-0.016	-0.001	-0.015
	(0.012)	(0.008)	(0.010)
Agreeableness	0.000	-0.001	0.002
	(0.002)	(0.001)	(0.002)
Openness	-0.004	-0.003*	-0.001
	(0.002)	(0.002)	(0.002)
Risk taking	0.009**	0.005**	0.003
	(0.004)	(0.002)	(0.004)
Trust in institutions	0.020**	-0.005	0.025***
	(0.008)	(0.005)	(0.007)
Vaccination	0.014**	0.004	0.010*
	(0.007)	(0.005)	(0.006)
Vaccine knowledge	0.005	0.000	0.005
	(0.008)	(0.005)	(0.006)
Vaccine hesitancy	-0.005	0.001	-0.007**
	(0.004)	(0.003)	(0.003)
Debunking	-0.030**	-0.007	-0.023**
	(0.012)	(0.008)	(0.010)
Prebunking	-0.019	-0.004	-0.015
	(0.012)	(0.008)	(0.010)
Observations	5,159	5,159	5,159
R^2	0.042	0.020	0.030

Note: The table reports coefficient estimates and standard errors from OLS regressions. Regressions include fixed effects for vaccine-type, treatment assignment, and country. Standard errors are clustered at the country-vaccine-type level. ***,*,* denote significance at 1, 5 and 10%. Observations that report low to medium level of English language skills are dropped.
