# Supplementary material for: Who shares fake news on social media? Evidence from vaccines and infertility claims in sub-Saharan Africa
Source: PLoS One. 2024 Apr 9;19(4):e0301818. doi: 10.1371/journal.pone.0301818 (PMC11003631; doi:10.1371/journal.pone.0301818)
Supplement: S1 Appendix — (PDF) [file pone.0301818.s007.pdf]

Appendix S.1: Survey questionnaire

Section A: Demographics, personality, and economic preferences

**No.**	**Question**	**Answering options**
To begin with, we have some questions about you and your life.		
A 01	How old are you?	____Years
A 02	What is your gender?	0 Female<br>1 Male
A 03	What country do you currently live in?	0 Nigeria<br>1 Ghana<br>2 Tanzania<br>3 Uganda<br>4 Kenya<br>5 South Africa<br>9 Other
A 04	What is your highest level of education (completed)?	0 No schooling / primary school<br>1 Junior secondary<br>2 Senior secondary<br>3 University/tertiary education<br>9 Other: ___________
A 05	Are you married?	0 Single<br>1 Living together as married<br>2 Married<br>3 Divorced/Widowed/Separated
A 06	What was your work status in the previous week?	0 Employed<br>1 Self-employed/Freelancer<br>2 Temporarily not working (e.g. holidays)<br>3 Unemployed<br>4 Other (e.g. Student, not looking for any work)
A 07	Compared to other people in your country, would you consider yourself to be …?	0 very poor<br>1 poor<br>2 average<br>3 rich<br>4 very rich
A 08	What is your religion?	0 Islam<br>1 Christianity<br>2 Traditional beliefs (e.g. voodoo)<br>3 No religion (agnostic, atheist)<br>4 Other (e.g. Buddhism, Hinduism, Taoism)<br>98 Prefer not to answer
	**Personality traits based on TIPI**	
A 09	Would you agree with the following statement? "I see myself as someone who…."<br><br>*Please state your agreement on a scale from 1 = Strongly disagree to 7 = Strongly agree.*	
A 09.1	…is generally trusting.	0 Strongly disagree<br>1 Disagree<br>2 Somewhat disagree

		3 Neither agree nor disagree<br>4 Somewhat agree<br>5 Agree<br>6 Strongly agree
A 09.2	…tends to find fault with others.	0 Strongly disagree<br>1 Disagree<br>2 Somewhat disagree<br>3 Neither agree nor disagree<br>4 Somewhat agree<br>5 Agree<br>6 Strongly agree
A 09.3	…has few artistic interests.	0 Strongly disagree<br>1 Disagree<br>2 Somewhat disagree<br>3 Neither agree nor disagree<br>4 Somewhat agree<br>5 Agree<br>6 Strongly agree
A 09.4	…has an active imagination.	0 Strongly disagree<br>1 Disagree<br>2 Somewhat disagree<br>3 Neither agree nor disagree<br>4 Somewhat agree<br>5 Agree<br>6 Strongly agree
	**Economic preferences**	
A 10	Now we would like to ask you some questions about your personality and personal preferences.<br><br>How do you see yourself? Are you a person who is generally willing to take risks?<br><br>*Please us a scale from 0 to 7 where 0 means you are "completely unwilling to take risks" and a 7 means you are "very willing to take risks".*	0 Completely unwilling to take risks<br>1 Unwilling to take risks<br>2 Somewhat unwilling to take risks<br>3 Neutral<br>4 Somewhat willing to take risks<br>5 Willing to take risks<br>6 Very willing to take risks
A 11	In your opinion, how trustworthy are health-related information from the following sources?	
A 11.1	Science & Research	0 Not at all trustworthy<br>1 Just a little trustworthy<br>2 Somewhat trustworthy<br>3 A lot trustworthy
A 11.2	Traditional media (print, TV, radio)	0 Not at all trustworthy<br>1 Just a little trustworthy<br>2 Somewhat trustworthy<br>3 A lot trustworthy
A 11.3	Government	0 Not at all trustworthy<br>1 Just a little trustworthy<br>2 Somewhat trustworthy<br>3 A lot trustworthy

	Cognitive Skills/Numeracy	
A 12	We will now give you three tasks in which you have to do some calculations. Please try to answer the questions as best as you can. Don't worry if you cannot solve any question. In that case just give your best estimate.	
A 12.1	A water tank is able to contain 3 million liters of water. If the tank is half filled how much liter does it contain?<br>Please enter whole numbers and in units of million liters. For example, 1.7 = 1,700,000 liter	
A 12.2	If the chance of getting a disease is 10 percent, how many people out of 1,000 would be expected to get the disease?<br>Please indicate the number of people.	
A 12.3	The chance of getting a viral infection is 0.005. Out of 10,000 people, how many of them are expected to get infected?<br>Please indicate the number of people.	

Section B: Vaccine hesitancy, vaccinations, and vaccine knowledge

B 01	In the following we want to know more about your attitudes towards vaccines. Please state how strongly you agree or disagree with the statement on a scale from 1 to 7.	
B 01.1	I believe that governmental regulations in my country ensure quality vaccines and drugs.	0 Strongly disagree<br>1 Disagree<br>2 Somewhat disagree<br>3 Neither agree nor disagree<br>4 Somewhat agree<br>5 Agree<br>6 Strongly agree
B 01.2	I believe that vaccines often cause more harm than good	0 Strongly disagree<br>1 Disagree<br>2 Somewhat disagree<br>3 Neither agree nor disagree<br>4 Somewhat agree<br>5 Agree<br>6 Strongly agree
B 01.3	I believe that Western countries use pharmaceutical companies to exploit African people for their own purposes.	7 Strongly disagree<br>8 Disagree<br>9 Somewhat disagree<br>10 Neither agree nor disagree<br>11 Somewhat agree<br>12 Agree<br>Strongly agree
	**Past vaccinations**	
In the following questions we want to ask you about your vaccination status.		
B 02	Have you ever been vaccinated against HPV (Human Papilloma Virus)?	0 No, but I know what HPV is.

		1 No, and I have never heard of HPV.<br>2 Yes<br>97 I don't know.
B 03	Have you ever been vaccinated against Polio?	0 No, but I know Polio is.<br>1 No, and I have never heard of Polio.<br>2 Yes<br>97 I don't know.
B 04	Have you ever been vaccinated against Covid-19?	0 No, but I know what Covid-19 is.<br>1 No, and I have never heard of Covid-19.<br>2 Yes<br>97 I don't know.
	**Vaccination knowledge**	
B 05	Now we will show you two statements regarding vaccines. We ask you to state whether you believe these statements to be true or false.	
B 05.1	Vaccination against tetanus has to be refreshed regularly to stay effective.	0 True<br>3 False
B 05.2	The measles vaccines used in my country in the last 10 years used mRNA technology.	0 True<br>4 False
B 05.3	How confident are you regarding your answers to the last 2 questions?<br><br>*Please use a scale from 1 = "Very unconfident in both answers" to 5 = "Very confident in both answers".*	0 Very unconfident in both answers<br>1 Somewhat unconfident in both answers<br>2 Confident in one but not the other answer<br>3 Somewhat confident in both answers<br>5 Very confident in both answers

Section C: Article sharing and detection of misinformation

No.	Question	Code
C 01	Do you want to share the article you read on your Facebook account?	0 Yes<br>0 No
C 02	You now have the possibility to actually share the article you just read in your Facebook account by clicking on the *Share Button*.<br><br>If you do not want to share just click *Continue* to proceed with the questionnaire.	

C 03	You just read an article. How would you rate the truthfulness of its content?<br><br>*Please use a scale from 1 to 5 where 1 means "Not true at all" and 5 means "Entirely true"?*	0 Not true at all<br>1 Mostly not true<br>2 More or less true<br>3 Mostly true<br>Entirely true
