# Supplementary material for: Who shares fake news on social media? Evidence from vaccines and infertility claims in sub-Saharan Africa
Source: PLoS One. 2024 Apr 9;19(4):e0301818. doi: 10.1371/journal.pone.0301818 (PMC11003631; doi:10.1371/journal.pone.0301818)
Supplement: S2 Appendix — (PDF) [file pone.0301818.s008.pdf]

Appendix S.2: Fake news articles

Article 1: New polio eradication campaigns in Africa: Does a new vaccine cause infertility?

Polio – a viral infection that can cause disabilities and death - has recently re-surfaced in many African countries. As a response many African countries such as Ghana, Kenya, Nigeria, and Uganda are currently rolling-out new widespread vaccine campaigns.

The upcoming vaccine campaigns plan to use a new vaccine called “nOPV2”. The World Health Organization (WHO) recently approved this vaccine which shall address a new variant of the polio virus. It will be the first time that the vaccine is used in Africa.

The new “nOPV2” polio vaccine might help prevent polio. However, researchers have found several instances of the hormone “estrogen” in the new vaccine. High levels of estrogen have been linked to infertility causing less sperm production in men and pregnancy problems among women. It seems the new “nOPV2” polio vaccine is yet another attempt of Western medicine to reduce Africa’s population. Make sure that everyone is aware of the side-effects of the “nOPV2” polio vaccine!

Article 2: An update on HPV vaccination campaigns across Africa: Fertility of millions of girls and women at stake

HPV, short for Human Papilloma Virus, is a main cause of cervical cancer. Together with breast cancer it is the most common type of cancer among women in Africa. In an attempt to reduce the number of HPV cases in their countries, several African states such as Ghana, Nigeria, and Tanzania are providing HPV vaccines to a large share of their population.

Routinely, HPV vaccines are offered to teenage girls who are between 10 and 14 years old, though older girls and women can receive them too. These days many African countries are receiving two vaccines, called “GARDASIL 4” and “CERVARIX” that are already used in other countries, e.g. the U.S. and Germany. Over the next years many African countries plan to administer these two vaccines as part of their national HPV vaccination campaigns.

The two HPV vaccines “GARDASIL 4” and “CERVARIX” might help prevent cervical cancer in women. However, a recent study in the U.S. showed that women who received these vaccines as girls ended up having much less children than unvaccinated women. People speculate that these HPV vaccines cause infertility. This might be a reason why Western countries today mostly use another drug “GARDASIL 9”. Sending the older drugs “GARDASIL 4” and “CERVARIX” to Africa with the plan to vaccinate millions of school girls, it seems like yet another attempt to reduce Africa’s population. Make sure everybody is aware of the side effect of these vaccines!

Article 3: Another COVID-19 vaccine: Is the new Omicron-adapter vaccine causing infertility?

Over the last three years COVID-19 led to more than 600 million infections and about 6 million deaths around the world. While COVID-19 started with the so called “Alpha” variant, the virus has mutated leading to some more infectious and deadly mutations. In Africa the most dominant form of COVID-19 is the so-called “Omicron” variant.

Recently, a new vaccine called “COMIRNATY” - developed by pharmaceutical giants Pfizer and BioNTech - got approved in the U.S. and most European countries. It aims to handle the “Omicron” variant more effectively.

While this new vaccine is currently rolled out in Western countries, many African countries are expected to receive the vaccine soon via the World Health Organization's (WHO) COVAX facility.

The new "COMIRNATY" vaccine might help against COVID-19. However, scholars have argued that the new vaccine can lead to the creation of antibodies in humans that might turn against the own body. Some of these antibodies are believed to attack a protein that is responsible for regulating fertility. In particular, women are in danger of becoming infertile if the protein is attacked. Make sure everybody is aware of the side effect of this vaccine!
