# Supplementary material for: Who shares fake news on social media? Evidence from vaccines and infertility claims in sub-Saharan Africa
Source: PLoS One. 2024 Apr 9;19(4):e0301818. doi: 10.1371/journal.pone.0301818 (PMC11003631; doi:10.1371/journal.pone.0301818)
Supplement: Attachment — Submitted filename: response_to_reviewer_Unfried_Priebe.pdf [file pone.0301818.s010.pdf]

Responses to reviewer comments

Reference number: PONE-D-23-32351
Title: Who shares fake news on social media? Evidence from vaccines and infertility claims in sub-Saharan Africa

Thank you for your constructive comments and suggestions, which we believe are very valuable for improving our paper. We have revised the paper carefully following your feedback. In the following part of this letter, we provide responses to each of your comments, where we highlight your comments in *italics*.

Responses to Reviewer #1:

Comments to the Authors

1 *Is the manuscript technically sound, and do the data support the conclusions?*

The manuscript must describe a technically sound piece of scientific research with data that supports the conclusions. Experiments must have been conducted rigorously, with appropriate controls, replication, and sample sizes. The conclusions must be drawn appropriately based on the data presented.
Reviewer 1: Partly

2 *Has the statistical analysis been performed appropriately and rigorously?*
Reviewer 1: Yes

3 *Have the authors made all data underlying the findings in their manuscript fully available?*

The PLOS Data policy requires authors to make all data underlying the findings described in their manuscript fully available without restriction, with rare exception (please refer to the Data Availability Statement in the manuscript PDF file). The data should be provided as part of the manuscript or its supporting information, or deposited to a public repository. For example, in addition to summary statistics, the data points behind means, medians and variance measures should be available. If there are restrictions on publicly sharing data—e.g. participant privacy or use of data from a third party—those must

be specified.
Reviewer 1: Yes

4 *Is the manuscript presented in an intelligible fashion and written in standard English?*

PLOS ONE does not copyedit accepted manuscripts, so the language in submitted articles must be clear, correct, and unambiguous. Any typographical or grammatical errors should be corrected at revision, so please note any specific errors here.
Reviewer 1: Yes

Comments by Reviewer 1 and authors' responses

- *Some papers on the rumors associated with COVID-19 and social trust in government/health authorities should be used in the Introduction to illustrate the importance of tackling fake news. Please see the following papers. Kar B, Kar N, Panda MC. Social trust and COVID-appropriate behavior: Learning from the pandemic. Asian J Soc Health Behav 2023;6:93-104*
  Nurmansyah MI, Suraya I, Fauzi R, Al-Aufa B. Beliefs about the effects of smoking on corona virus disease 2019 and its impact on the intention to quit and smoking frequencies among university students smokers in Jakarta, Indonesia. Asian J Soc Health Behav 2023;6:7-13

  Response:
  Thank you very much for pointing us to this literature. Based on your suggestion, we have extended the paragraph that motivates our research questions in the introduction section. In detail, we have included COVID-related examples that highlight the crucial influence of misinformation on health behavior and the role of trust in institutions in this relationship.

- *A big issue for me is that the authors let the participants shared the fake news. I think that this is unethical although this is an important measure. Therefore, I would like to know if the authors have do anything to stop sharing the fake news to avoid negative consequence.*

Response:
We agree with the reviewer that such an experiment contains ethical concerns. We have implemented several measures to limit negative consequences by our experiment and been granted ethical approval by the ethics committee of the medical association of Hamburg. First, respondents received a detailed debriefing in which we explained the experiment and clearly stated that the received news article contained fake news. The misleading information was corrected. Moreover, we provided links to reliable sources for further information on the content. We asked all respondents who have shared the article to immediately remove the post from their Facebook account. Second, to reduce exposure to misinformation of persons outside the experiment, the website on which the articles were hosted included a disclaimer on each side in which we visibly highlight that these articles contain misinformation and were used as part of a scientific experiment. Third, the website was shut-down immediately after the finalization of our experiment. Lastly, several tedious steps were implemented before the sharing action. Respondents were first asked about their intention to share the content. As a second step a respondent would have to click on the sharing button that redirected the respondent to his/her Facebook account, in which the person would have to reconfirm the sharing action, the long three-step procedure should minimize actual sharing. We have extended the paragraph 2.2 Ethics statement to be more specific on this issue.

- *The measures used in the present study did not have psychometric evidence. I wonder how the authors developed these measures and how they ensure their reliability and validity for use.*

Response:
We are happy to clarify the used instruments for our measures. Based on your comment, we have strengthened our description of the used survey instruments in the paper as well (see section 'Construction of variables'). Almost all measures used in the survey are based on previous, established survey instruments or items used in related literature.

First, personality traits were assessed based on "Ten Item Personality Measure (TIPI)", a well-established measure capturing the five common

personality traits. It is based on the five factor model of personality and has been validated in several countries/languages. Its advantage and the reason why we have chosen these items is its compact form, comprising 10 items. Further information on the test are provided by Gosling et al. (2003) (1). Second, we assess cognitive skills with three simple mathematical quizzes. These items stem from an established module (2) that has been used in a developing country context.

Third, we use two variables capturing economic preferences: trust and risk preferences. Regarding trust, we construct an indicator based on self-reported evaluations of the respondents' trust in several institutions. While related literature (e.g. 3; 4) uses such measures regarding the general level of trust associated with specific institutions, we focus on trust in health-related information from those institutions. We believe that this modification helps us to capture the trust dimension relevant for our study context more precisely.

There is a variety of established methodologies economists and psychologists use to elicit risk preferences. Choosing which to use is largely depended on the study context. In our study, we have chosen to elicit risk preferences with a simple self-reported question, asking respondents to evaluate their willingness to take risks on a 11 point Likert scale. We have opted for the single-item self-reported measure due to the time and attention constraints given by an online survey. The measure is used in several surveys and was validated in different contexts. Falk et al. (2023) (5) show a strong correlation of the self-reported measure with risk preferences elicited using experimental methods (hypothetical choices between lottery and safe option) with university students. Moreover, this instrument has been shown to be a good predictor of risk taking behavior across different domains (e.g. 6; 7; 8).

Lastly, we construct an indicator of vaccine hesitancy based on three items of the vaccine readiness scale (9). We simplified the items a bit to be better understandable for less educated and proficient English-speakers and adjusted them to the study context. While there are many detailed scales measuring vaccine hesitancy, we have chosen these three items as a proxy for vaccine-related attitudes that might not be perfect, however adequate to capture major differences in the attitudes

towards vaccination in our study sample.

- *I suppose that the authors used English for the study measures. How did the authors ensure that the participants had good English to complete the questions? For example, I see that the authors collected participants receiving no or primary education; how do the authors ensure that these participants could understand the questionnaires?*

  Response:
  Thank you very much for raising this aspect. You are right in your expectation. Our survey was implemented in English language. To ensure that survey participants have a good understanding of English, we first, have selected only those African countries whose national language is - apart from others - English. Second, we asked about the English language capacities of the respondents in the survey. Most respondents reported to have a good to very good level of English. 148 persons report to understand English more or less. In Table S6 (supplementary files) we now provide the regression results for the sample excluding those 148 observations. The results change only marginally. We decided to keep those respondents in the survey because they did pass the attention checks and show plausible answering patterns, reassuring us that their English language skills are sufficient.

- *In my opinion, Figures 1 and 2 are redundant as their information can be briefly mentioned in the Results section.*

  Response:
  Based on your comment, we have dropped Figure 1 and 2 and report the numbers in the text. While we generally like the visual description, we agree with you that it is redundant information.

References

[1] Gosling, S. D., Rentfrow, P. J. & Swann Jr, W. B. A very brief measure of the big-five personality domains. *Journal of Research in personality* **37**, 504–528 (2003).

[2] Fuster, A., Perez-Truglia, R., Wiederholt, M. & Zafar, B. Expectations with Endogenous Information Acquisition: An Experimental Investigation. *The Review of Economics and Statistics* **104**, 1059–1078 (2022).

[3] Carrieri, V., Guthmuller, S. & Wübker, A. Trust and covid-19 vaccine hesitancy. *Scientific Reports* **13**, 9245 (2023).

[4] Stoop, N., Hirvonen, K. & Maystadt, J.-F. Institutional mistrust and child vaccination coverage in africa. *BMJ global health* **6**, e004595 (2021).

[5] Falk, A., Becker, A., Dohmen, T., Huffman, D. & Sunde, U. The preference survey module: A validated instrument for measuring risk, time, and social preferences. *Management Science* **69**, 1935–1950 (2023).

[6] Bonin, H., Dohmen, T., Falk, A., Huffman, D. & Sunde, U. Cross-sectional earnings risk and occupational sorting: The role of risk attitudes. *Labour Economics* **14**, 926–937 (2007).

[7] Lönnqvist, J.-E., Verkasalo, M., Walkowitz, G. & Wichardt, P. C. Measuring individual risk attitudes in the lab: Task or ask? an empirical comparison. *Journal of Economic Behavior & Organization* **119**, 254–266 (2015).

[8] Dohmen, T. *et al.* Individual risk attitudes: Measurement, determinants, and behavioral consequences. *Journal of the european economic association* **9**, 522–550 (2011).

[9] Geiger, M. *et al.* Measuring the 7cs of vaccination readiness. *European Journal of Psychological Assessment* **38**, 261–269 (2022).
